# Drug delivery to and through the skin

**DOI:** 10.1007/s13346-024-01614-w

**Published:** 2024-06-05

**Authors:** Richard H. Guy

**Affiliations:** Department of Life Sciences, University of Bath, Claverton Down, Bath, BA2 7AY U.K.

**Keywords:** Drug delivery technology, Topical and transdermal drug delivery, Skin barrier function, Skin penetration enhancement – chemical, Skin penetration enhancement – physical, Topical formulations and metamorphosis

## Abstract

Drug delivery technology has advanced significantly over >50 years, and has produced remarkable innovation, countless publications and conferences, and generations of talented and creative scientists. However, a critical review of the current state-of-the-art reveals that the translation of clever and sophisticated drug delivery technologies into products, which satisfy important, unmet medical needs and have been approved by the regulatory agencies, has - given the investment made in terms of time and money - been relatively limited. Here, this point of view is illustrated using a case study of technology for drug delivery into and through the skin and aims:  to examine the historical development of this field and the current state-of-the-art;  to understand why the translation of drug delivery technologies into products that improve clinical outcomes has been quite slow and inefficient; and  to suggest how the impact of technology may be increased and the process of concept to approved product accelerated.

**Graphical Abstract:**

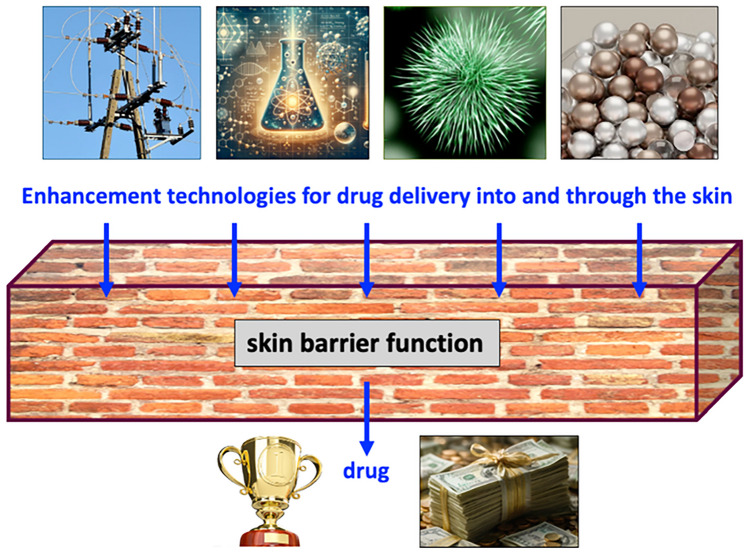

## Introduction

A useful starting point for any objective consideration of drug delivery in general has been captured in an excellent quote (see **X**, formerly known as Twitter, October 16, 2023) from Samir Mitragotri, an internationally recognised leader in the field: *“Drug and Delivery are [the] ‘what’ and ‘how’ of therapeutic products. Without knowing ‘what’, ‘how’ is irrelevant. And without answering ‘how’, ‘what’ is not possible.”* The irrefutable corollary to this statement is that until a drug is delivered properly, it can never be the active ingredient of an approved medicine.

With respect to drug delivery into and through the skin, there are a number of issues worthy of examination. First and foremost is a question that one often reads at the start of many publications addressing the subject; specifically, why is it so difficult to entice topical/transdermal drugs to reach their therapeutic targets within, just below or beyond the skin? The answer of course is because the skin is a formidable biological barrier to drug entry and no endogenous mechanism other than simple, passive diffusion is available to overcome this challenge [1]. The principal element of the barrier is the stratum corneum (SC), the composition and structural organisation of which are key to its function [2]. Indeed, the SC provides a physical resistance to chemical transport both from within the body to the external environment (its evolutionary *raison d’être* for water retention) and from the skin surface into and through the skin (as for topical/transdermal drugs) [3]. The mortar-and-brick visualization of the SC – intercellular lipids providing the ‘cement’ around the terminally differentiated corneocyte ‘bricks’ – offers little in terms of free volume for the diffusion of anything other than small molecules [4]; while a cut-off of 500 Daltons is often cited in the literature [5], it is clear that the resistance to compounds larger than this falls off rather steeply thereafter.

The key role of the skin begs the next question, therefore, as to why so much effort has been devoted to developing technologies that can overcome the difficulty associated with drug delivery to and through this exceptional biomembrane? Obviously, for drugs that locally treat dermatological disease, it makes much more sense to apply the medicine directly to the affected site where the target is so close, rather than administering a much larger amount of the active (by injection or orally, for example) to ensure adequate cutaneous bioavailability. The case for using the skin route for drugs that act systemically, on the other hand, is less easily made but – as discussed in more detail below – for the right compounds with the right physicochemical and pharmacokinetic properties, transdermal delivery can be extremely attractive [6].

The success of the transdermal patches, however, led to some poor expectation management. This drug delivery technology was perceived correctly as relatively simple and its essentially non-invasive and therefore patient-friendly nature made many then ask *‘well, why can’t we deliver more drugs by the skin route?’* As a consequence, multiple and progressively more complex approaches have been investigated, most with the aim of delivering more and, in particular, larger compounds (with insulin a favourite candidate) than the low molecular weight drugs that have been incorporated into transdermal patches. While this is, and remains, a laudable goal, it is here that translation has been problematic, as will be addressed with several examples later on.

In terms of drug delivery and formulation design for the local treatment of dermatological diseases, the level of innovation has (with a few notable exceptions) been modest… primarily, it appears, because many commonly used actives are very potent, and the quantities required to be absorbed for therapeutic effect are quite small [1]. As a result, satisfactory clinical outcomes can be achieved with only a few percent of the applied drug dose being absorbed. Apart from poor drug utilisation, this situation gives rise to some systemic safety concerns when such potent substances are applied to the skin of infants and small children or inadvertently to particularly permeable skin (e.g., when the barrier is disrupted) [7]. There is also a problem, in certain parts of the world, with access to dermatologic medicines as the mediocre performance of an innovator product creates problems for generic competition which, of course, must match the lacklustre standard to prove its bioequivalence (that, in the main, has required costly, insensitive and prolonged clinical trials). More recently, however, regulatory initiatives – particularly from the US Food & Drug Administration (FDA) and the European Medicines Agency (EMA) [8, 9] – have encouraged the development of alternative approaches for the assessment of cutaneous bioavailability [10, 11] and for the characterisation of topical formulations and their transformation to a residual film upon application to the skin. This has opened a door for greater creativity to be brought to bear on formulation design and performance optimisation, as outlined at the end of this paper.

## Transdermal drug delivery systems – a success!

Not so long ago, it was not uncommon to hear statements about the transdermal route of administration suggesting that this approach may not be such a great idea; for example: *“this is a relatively minor subject, not worth the time to discuss; anyway, skin is too good a barrier and makes the route of administration unfeasible; and everyone knows that transdermal delivery will never be possible for biotech drugs.”* Today, this pessimistic view can be countered with facts that are now broadly accepted [6]:

- Transdermal delivery is a successful controlled release technology;
- The transdermal route is extremely attractive for the right drugs;
- The transdermal route is completely unfeasible for others; and
- Novel transdermal technology (with microneedles a current front-runner) may well enable topically applied macromolecular drugs to reach targets within and beyond the skin.

**Fig. 1:**
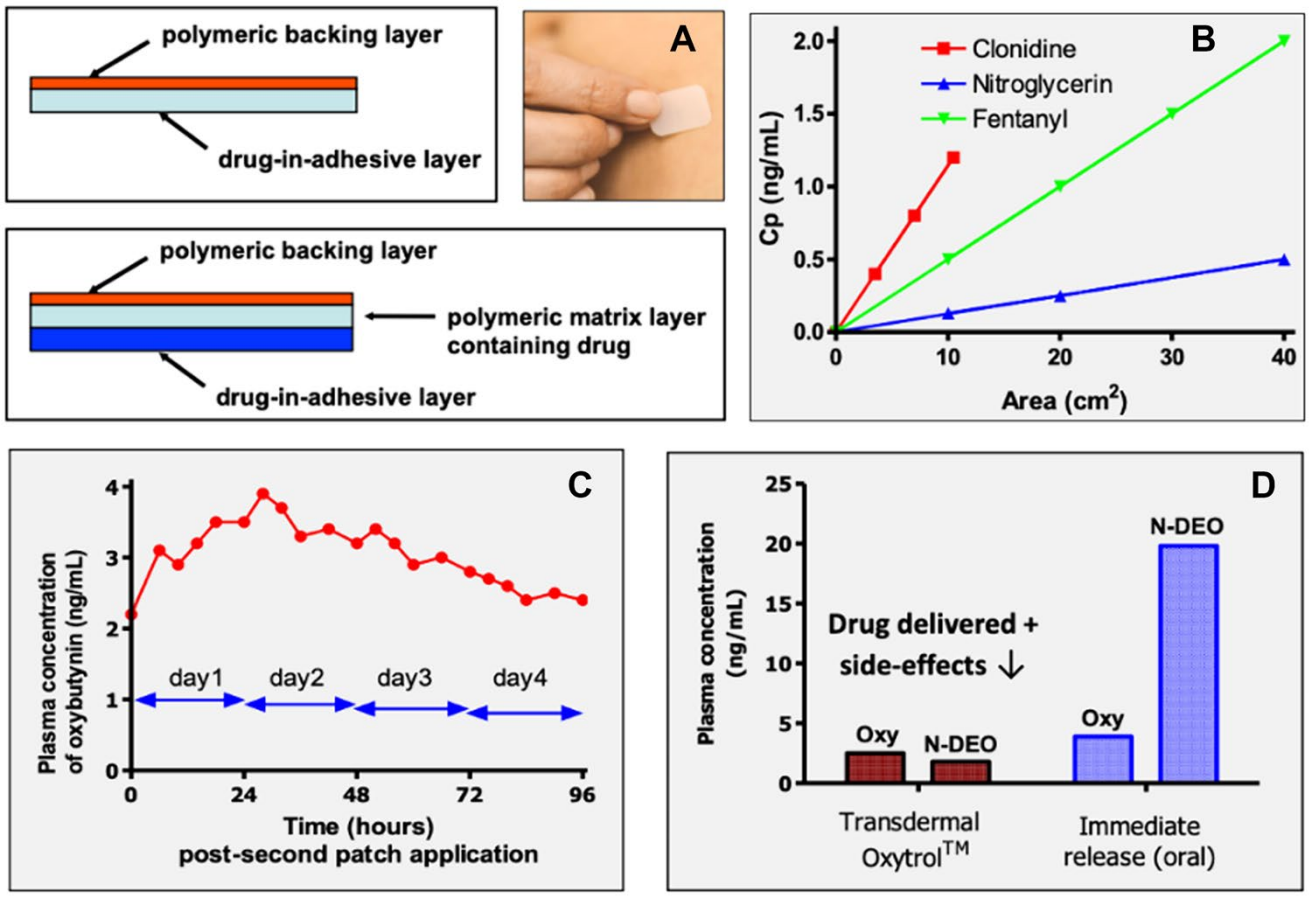
**A** Common transdermal patch designs used in marketed systems. **B** Demonstration of how patch area can control and determine drug input across the skin. **C** Close to zero-order transdermal delivery of oxybutynin over a 3-day period from a single patch. **D** Clear demonstration of avoidance of hepatic first-pass effect when oxybutynin is administered transdermally

The rationale for transdermal delivery as it is known today originated from the fact that the topical application of classic drug formulations, such as a cream or ointment, for systemic delivery (e.g., of nitroglycerin in the treatment of angina) was inefficient and inelegant. The poor control of dose and area of application resulted in large variations in the extent and duration of drug effect. The solution to this shortcoming was the transdermal patch pioneered by the Alza Corporation in Palo Alto (California) that presented the drug to the skin from a delivery system, the design and area of which permitted drug input into the systemic circulation at a controlled and predictable rate (Fig. 1A, B) [6, 12, 13]. Modern-day patches are thin, simple in structure and discreet, and permit (typically zero-order) drug delivery for periods of 0.5 to 7 days (Fig. 1C).

The established advantages of transdermal delivery are well-known [12, 13]: pre-systemic (first-pass) metabolism can be circumvented allowing smaller daily doses to be used and favourably changing metabolite profiles (Fig. 1D); drug levels can be maintained within the therapeutic window for a prolonged period, extending the duration of drug action and reducing the frequency of dosing; as a result, inter- and intra-patient variability may be reduced and, overall, improved patient compliance and acceptability of drug therapy can be achieved; finally, and of practical value, drug input can be terminated simply by patch removal.

But, as inferred above, suitable drugs for transdermal delivery must satisfy some stringent criteria, including (most importantly) high potency to ensure a patch area of acceptable size, low molecular weight, lipophilic (but not excessively so), unfavourable pharmacokinetics when administered orally (e.g., short half-life, inconvenient dosing), and skin-friendly (i.e., not irritating or sensitizing) [6]. Yet, despite this high bar, transdermal drug delivery via passive diffusion from patches has proved to be a commercial success with more than 20 approved compounds and multiple products on the market (including combination products and generics) [6]. There is no question that transdermal delivery is now a mature technology, with the current, annual global market approaching US$ 8B.

## Chemical enhancement of transdermal drug delivery: great science ≠ successful translation

The early success of transdermal patches, coupled with the realisation that the pool of sufficiently potent drugs, for which this route of administration may be feasible, was quite limited, encouraged a large and ongoing effort to identify technologies for increasing molecular flux into and across the skin. The quest for chemical penetration enhancers has probably accounted for more research time and investment – and to be fair, publication numbers (including whole books [14–16]) – than any other approach but has foundered because those compounds able to effectively decrease skin barrier function are also local irritants [17]. Indeed, the correlation between degree of stratum corneum perturbation with skin irritation is generally acknowledged.

**Fig. 2:**
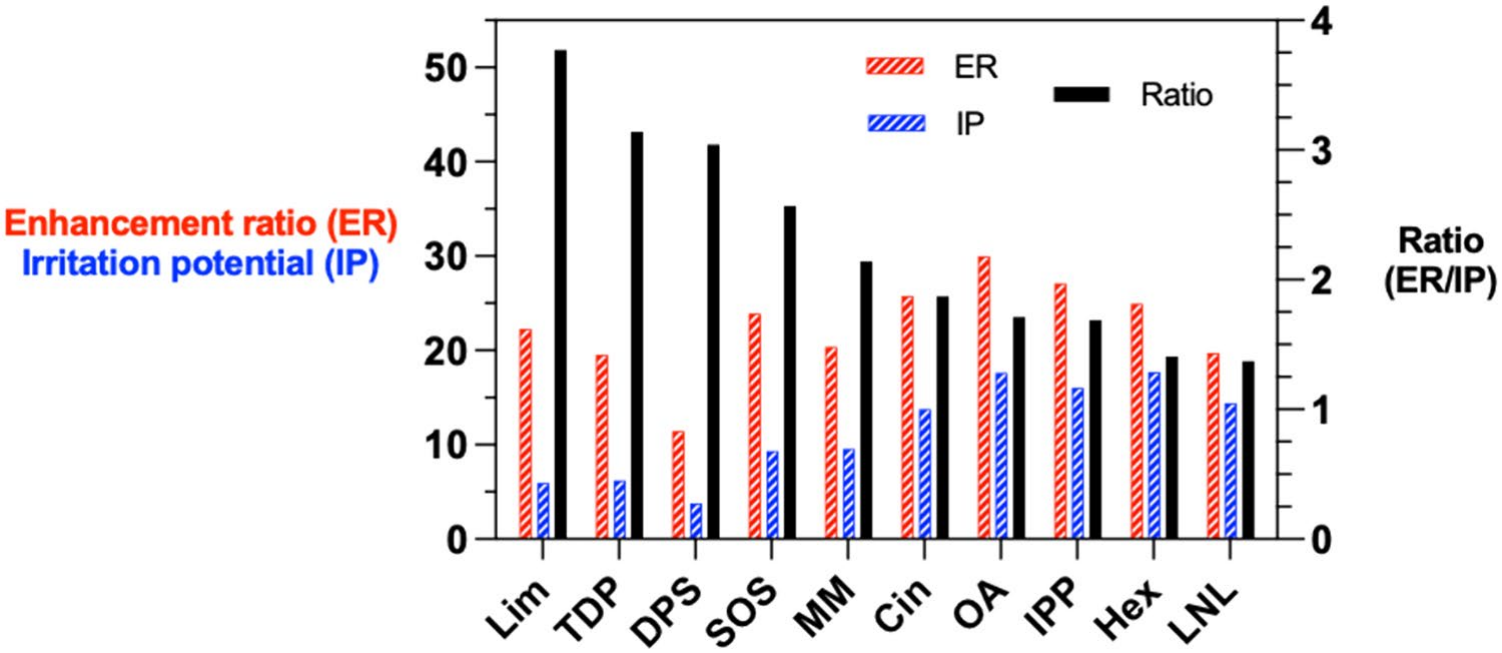
Indirect measurements of skin penetration enhancement (ER) and irritation potential (IP) are, in general, correlated (data redrawn from [18]). Searching for the ‘sweet spot’ where ER/IP > 1 has yet to bear significant fruit

With hindsight, and despite creative efforts to find the elusive ‘sweet spot’ (Fig. 2) [18], this observation is far from surprising since disruption of the skin’s barrier function, by whatever means causing the effect, immediately sets in motion an alert that something is amiss (i.e., visual reddening/swelling of the skin) and a physiological process - such as restoring the composition and integrity of the SC intercellular lipids - to correct for the ‘damage’ that has been done.

Disappointment in the enhancer field has been compounded by so-called *promising* studies reporting large effects of certain chemicals on the permeability of rodent skin, which has long been recognised as a poor model for the human barrier [19]. There have also been false hopes raised by the first reports of impressive enhancement produced by relatively new chemical entities – Azone (1-dodecylazacycloheptan-2-one) providing perhaps a particularly good example [20]. The problem with novel excipients like Azone that do not appear on the list of GRAS materials – that is, “generally-regarded-as-safe” - is that regulatory agencies can view them as new chemical entities and demand, as a result, stringent criteria to be met to demonstrate their safety. Unsurprisingly, there has been reluctance to take on the associated financial burden for such studies and, as a consequence, the number of non-GRAS materials available for use in topical/transdermal drug delivery is vanishingly small.

Despite this rather disappointing outcome, this has not deterred some lateral thinking in the chemical enhancer space and there have been attempts to borrow the approach of drug association to cell-penetrating peptides for skin permeation enhancement. Initial efforts, reported in some high-profile publications 10–15 years ago, appeared to demonstrate feasibility for the idea and its application not only to small molecular weight drugs, but also to some macromolecules of different physicochemical properties and size [21, 22]. However, despite this success, the number of follow-up reports have dwindled, and no further translation of the concept has taken place.

## Iontophoresis – mature and proven technology still waiting for the “home run”

**Fig. 3:**
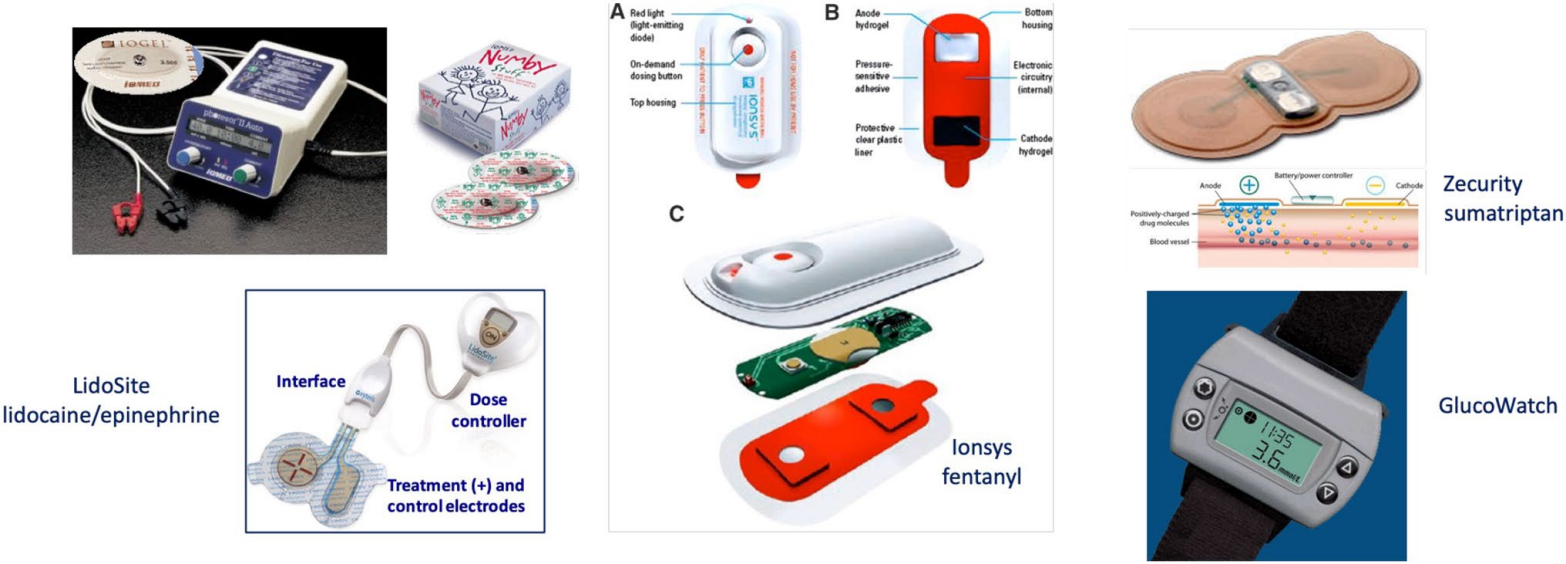
Commercialized iontophoretic drug products. LidoSite, Ionsys and Zecurity delivered drugs for local anaesthesia, the treatment of chronic and breakthrough pain, and migraine, respectively. The GlucoWatch, in contrast, used ‘reverse’ iontophoresis to transdermally extract (and the device then detected) glucose for the noninvasive monitoring of blood sugar

The application of a small direct current – either continuously or in a pulsatile fashion - to enhance and control the penetration of (typically) charged molecules across the skin is a technology that has been known and studied for many years [23]. The science base for iontophoresis is therefore well-established and real products have been developed, received regulatory approval, and commercialized (Fig. 3).

That having been said, the products which reached the market are no longer available for sale and it is appropriate to address why this is the case. For sure, the technology is a step-up in complexity and cost compared to a simple, passive transdermal patch. It follows that the medical application of iontophoresis, in terms of unmet need, has to outperform existing technology – in terms of one or more of patient/healthcare provider use, safety and efficacy, and price (to the patient or payer) - if commercial success is to follow. Further, while the lidocaine/epinephrine device was an impressive demonstration of how iontophoresis could be cleverly used to quickly induce and then sustain local anaesthesia, it did not exactly catch the imagination of big pharma and promise the next “big thing”. As a result, quite a lot of effort was (mis-)directed towards unfeasible applications of iontophoresis, with insulin delivery being perhaps the most egregious example.^1^

There clearly remain sensible uses of iontophoresis in the conventional and ‘reverse’ configurations [24], but their evolution will require both convincing business models to generate the necessary investment and sufficiently lucrative markets to justify the investment of time and resources in this proven drug delivery technology.

## Poration technologies – short-circuiting the stratum corneum (SC)

Frustration with the seemingly impenetrable nature of the SC inevitably led to the investigation and, at least initial, development of technologies to bypass the challenge by creating new, low-resistance pathways (also known as ‘holes’) through the barrier. In no particular order, these methods include physical/mechanical abrasion, electroporation, sonoporation, thermal and laser ablation [25–30] and, of course, microneedles [31] (which are discussed in a separate section below).

The primary motivation for this more drastic approach, of course, was the desire mentioned already to use the topical/transdermal route for the delivery of drugs of any size (including big ones). As all these poration technologies create openings in the barrier of micrometre or larger dimensions, just about any biopharmaceutical of interest can theoretically access the pathways created.

Unsurprisingly, porated skin turns out to be a lot more permeable to a lot more compounds but, equally predictably, the ‘price’ for this enablement is considerable. While some approaches might be labelled ‘minimally invasive’, others are clearly associated with pain, heat and sometimes even bleeding; all come with degrees of skin irritation of varying intensity. The devices themselves – with few exceptions – are far from discreet and typically quite expensive. The technologies invariably demand separate delivery systems (patches or formulations) and, in some cases, large-ish power sources to elicit the necessary poration of the SC.

Among the approaches considered, identification of the drug/disease target appeared to play a secondary role to showcasing the technique and demonstrating its ability to deliver a wide range of different compounds. However, it was not possible to escape the fact that, even with the SC barrier compromised, the transdermal route remains feasible for only a limited number of potent drugs; yes, the number is bigger than that for which passive delivery works, but it is not massively greater. As a result, each of the poration technologies has enjoyed a ‘moment in the sun’, with high-profile papers, intellectual property patented, new companies created and investment acquired, but none have yet reached the finishing line of a product translated and approved for clinical use.

### Microneedles… finally, the breakthrough?

Of all the ‘poration’ technologies that have been developed, microneedles (MNs) have proved to be the most tenacious and, in terms of numbers (for the moment, at least), they dominate all else in the transdermal/topical peer-reviewed literature and in conference presentations, posters, etc. The claimed advantages of this approach are well-known [31] and include by-passing the skin barrier, accurate dose control, minimally invasive (‘sensation-less’), single-use/dose, disposable, enhanced drug stability (no cold chain required, for example), reduced or no sharps waste, reduced patch wear time, easy to apply, smaller packaging and lower manufacturing costs compared to needle-and-syringe, and potential to target sites in the skin. Taken together, these positive features are suggested to collectively permit more efficient and faster drug delivery, improved vaccine immunogenicity, enhanced drug access socio-economically, increased safety (via cost savings), and improved patient compliance.

**Fig. 4:**
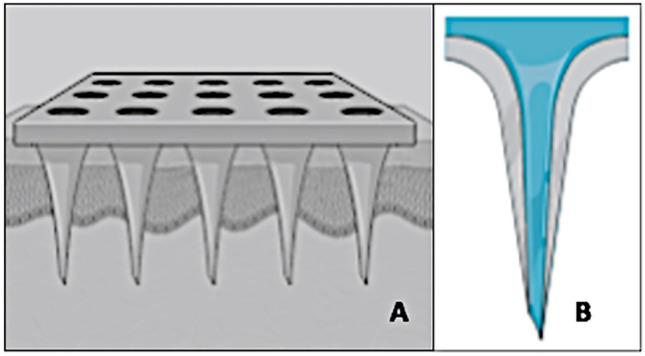
**A** Schematic diagram of the microneedle ‘concept’, enabling the active pharmaceutical ingredient to access the viable layers of the skin without having to overcome the substantial barrier of the stratum corneum. **B** Expanded view of the ‘hollow’ type of microneedle. (Created with BioRender.com)

MN technology (Fig. 4A) embraces several design options [32] that can be broadly categorised as follows: (a) solid MNs are inserted and removed, followed by topical drug application (in the form of a patch or semi-solid formulation, for example), (b) hollow MNs post-insertion allow drug solution to flow into the skin from a supply on the surface until removal (Fig. 4B), (c) dissolving MNs after insertion hydrolyse and release drug to the point of complete degradation, and (d) swellable MNs, once inserted, absorb interstitial fluid creating an aqueous hydrogel from which drug is then released.

Yet, despite the sound rationale for MN-mediated drug delivery, the increasing maturity and diversity of MN designs available, more than 20 years of research on an astonishingly broad range of potential drug candidates of all ‘flavours’ and sizes, and some serious attempts to advance product development to regulatory approval, the wait for the first commercialised MN-based medicine continues.

So, why is this and where is the field today [31, 32]? It was recognised early on in the MN era, that vaccines represent the low-hanging fruit for this approach to drug delivery: the required doses are small, the therapeutic windows are broad, the skin is a biologically beneficial route of administration for vaccination, and the acute nature of vaccine administration alleviates many safety concerns relative to those associated with chronic dosing regimens involving repeated MN insertions over potentially very long periods of time. This assessment has been borne out by the successful demonstration in both animal models and human volunteers that immunisation to a number of viral diseases can be achieved using MN-based patches [33–35]. It seems reasonable to anticipate, therefore, that regulatory acceptance of a MN-administered vaccine product will be forthcoming in the not-too-distant future, and it appears that a combined measles-rubella vaccination is leading the way [36].

While it is probable that other vaccines will follow, there remain obvious challenges for drugs that must be dosed repetitively, and this has been borne out recently by unsuccessful regulatory outcomes for MN patches designed to deliver the anti-migraine drug, Zolmitriptan, and the synthetic peptide (34 amino acids), Abaloparatide, for anabolic osteoporosis treatment. In both cases, clinical endpoints were missed suggesting that delivery objectives were not achieved. The way forward, in terms of expanded translation of the promise of MN-based drug delivery, has to address and optimise (at least) (i) the repeatability and consistency of MN insertion into the skin (and the means to ensure this), (ii) the safety of long-term application of MN drug delivery systems, and (iii) methods of affordable manufacturing.

## Nanoparticles and other “-*somes*”

To the best of my recollection, the idea that nanoparticles might in some way enhance drug delivery into and/or through the skin originated with work using liposomes in the 1980s [37, 38]; over subsequent decades, the same capability has been reported for a variety of colloidal systems, including (but not limited to) ethosomes, transfersomes, and niosomes [39–42]. Another wave of formulations then arrived in which the ‘carriers’ were more often referred to as nanoparticles (replacing the now, apparently old-fashioned *“-somes”*) or other nano-structures, such as solid lipid nanoparticles and even quantum dots!

The literature is now awash with publications claiming successful demonstration of the magic of the word “nano” [43]. Much of this effort, however, has failed to provide any tangible evidence that the particles themselves have played any role in the results observed. While it is plausible that the components used to form the colloidal structures involved may act as skin penetration enhancers (via, for example, lipid structure perturbation), the fanciful idea that the nano-things themselves are bludgeoning their way across the stratum corneum and popping out the other side with drug load still safely on-board, so to speak, remains doggedly unproven.

So, what is known? On the positive side for nano-enthusiasts, reasonably good data from different sources have shown that particles – some even more micro than nano – can accumulate at the openings and beyond of hair follicles and remain in place for periods of time during which drug can be released and taken up [44]; again, however, there is scant evidence for these particles ever ‘escaping’ from the follicles by any pathway other than the one used to enter it in the first place. As indicated above, some particles can provide a source of (typically) surfactant-like compounds that resemble known penetration enhancers and produce improved drug delivery. The most impressive results from this ‘mechanism’ have been seen, of course, in rodent skin and most often without further verification in a more useful animal model.

**Fig. 5:**
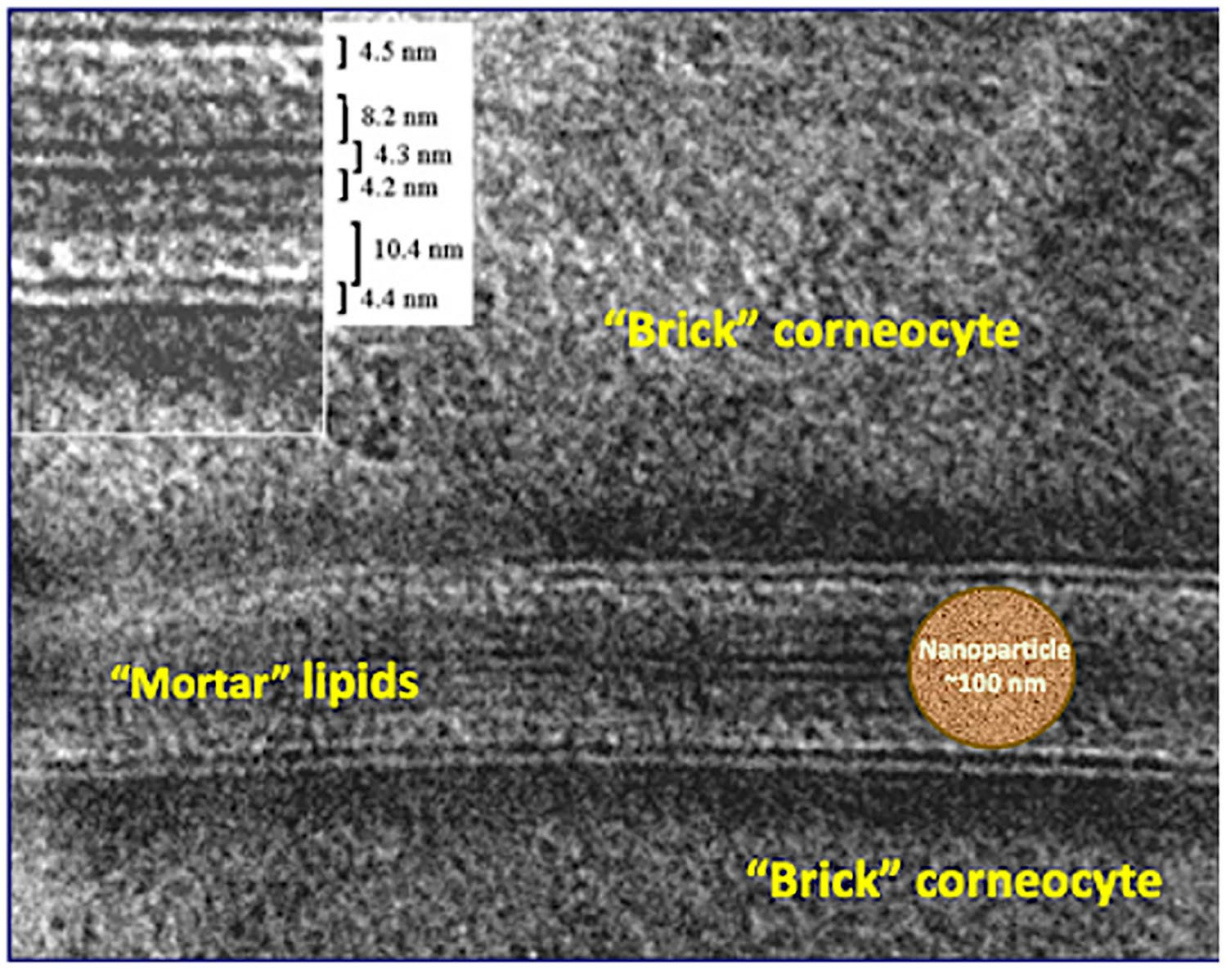
The stratum corneum is a “brick wall” (10–20 µm thick) with keratin-filled bricks about 0.5 µm across. The space between corneocytes (25–100 nm) is filled with lipid lamellae. How does a 100 nm (or bigger) particle diffuse across this barrier? The micrograph was kindly provided by Dr. Lars Norlén

Less inspiring is the frequent absence of any attempt to account for observations of nanoparticulate success as drug carriers across the skin, and a reluctance to note that such remarkable results are inconsistent with (“boring!”) experiments showing quite unequivocally that, when one puts particles on the surface of the skin, they really do not go anywhere until they are washed off [45–47]. The authors of such studies have – essentially, without exception – failed to explain how these particles have accessed the necessary free volume in the stratum corneum to enable their diffusion across the barrier (Fig. 5).

To sum up, the application of nanoparticles (of any stripe) to the skin has not resulted in any approved medicinal products that acknowledge the essential role of the colloidal element in their function. The cosmetic industry, on the other hand, has profited significantly by the incorporation of various *“-somes”* and other nano-structures…

## Topical drug delivery technology – signs of progress

It is fair to say that the majority of approved and marketed topical formulations under-perform in terms of delivery efficiency; the percentage of an applied topical drug dose that is actually absorbed to its target in the skin rarely exceeds 5% and is frequently much less than that. The fact that a large number of products are, nevertheless, commercially available and, in large part, safe and effective, is a reflection of the often-potent nature of the active pharmaceutical ingredients. As a result, the incentive for innovation in dermatological formulations has been low and new ideas have been limited.

An important reason for the poor delivery of drugs from topical products is that the change in the properties of the formulation as it is rubbed into the skin have not been fully considered with respect to the impact on uptake [1, 7, 48]. As key excipients are absorbed themselves, or lost via evaporation (if volatile), the drug’s solubility in the transforming residual phase is undermined to the point that it may no longer be soluble in the components remaining on the skin surface (leaving the active stranded on the skin surface in the solid state) and absorption therefore stops. This ‘metamorphosis’ means that a significant amount of the drug applied is wasted and that permeation is poor and variable.

**Fig. 6:**
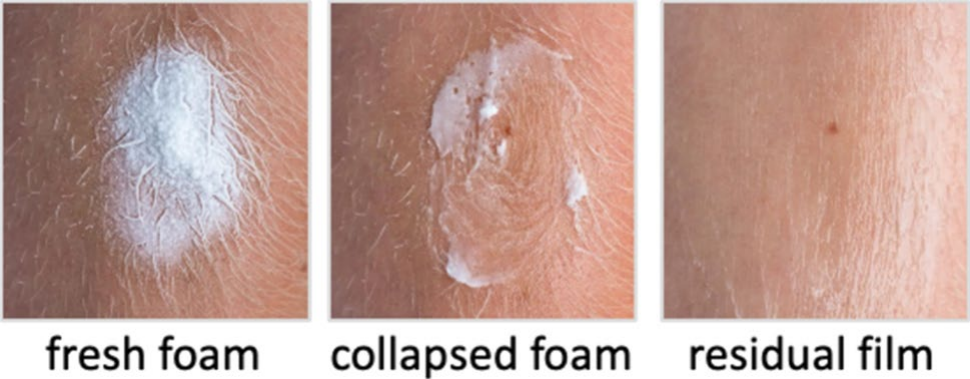
Metamorphosis of the calcipotriene and betamethasone dipropionate foam. From the solution in the manufactured product, in which the drugs are dissolved, and upon application and collapse of the foam, the drug’s attain their saturation solubilities (and maximum diving forces for skin penetration), and then create a residual ‘ointment’ film where a transient period of supersaturation may occur and in which thereafter finite amounts of the drugs remain in solution (at saturation) to sustain delivery

Recognition of this issue has resulted in new thinking, in terms of a formulation strategy, with more focus now on designing not only the product to be manufactured and packaged, but also to optimising the residual phase post-application to create a delivery system that continues to function as such after the ‘metamorphosis’ has occurred [1, 7, 49]. A notable example of this approach is a calcipotriene (0.05 mg/g) and betamethasone dipropionate (0.5.mg/g) foam (Enstilar^®^, Leo Pharma), to treat psoriasis vulgaris in adults that was approved by the FDA in 2015 (Fig. 6).

The other positive development in topical drug delivery is that methods to assess cutaneous bioavailability have been subject to increased focus in recent years. This not only provides a simpler path towards the assessment of bioequivalence between a less expensive generic drug product and the reference formulation, but it also means – in combination with the realisation that product development needs to optimise the residual film post-application as well - that innovative and superior formulations with lower drug loads that deliver a greater percentage of the administered drug dose can be created.

As inferred above, this initiative is benefiting from a regulatory science imperative to continue and refine validated, surrogate tests to objectively evaluate cutaneous pharmacokinetics, with the objective to develop better tools to establish bio(in)equivalence between drug products and to provide a faster and cheaper route-to-market for generic medicines and improved patient access to more affordable healthcare.

## Take-home messages

- Transdermal drug delivery from conventional patches is a mature and commercially successful technology.
- The marriage of physical techniques with topical/transdermal delivery has been bumpy and no truly breakthrough products have emerged to-date.
- Research and development into microneedle-based drug delivery into and through the skin remains buoyant, with vaccination applications in the vanguard, but further challenges await future translation of this technology.
- Skin barrier function remains formidable: chemical enhancement has been constrained by skin irritation; (nano)particle-based approaches have not delivered.
- Topical product innovation, research into new methods to assess cutaneous bioavailability and improved accessibility to affordable medicines are “on the up”!

## Data Availability

No new data are presented in this article.

## Footnotes

1 U.S. patent number 5,843,015, granted December 1, 1998, “Molecules for iontophoretic delivery”, co-invented by R.A. Hoke, and B.H. Sage, Jr., spells out clearly those peptides/proteins that are – and, importantly, are not (insulin being one example) – viable options to consider for iontophoretic delivery.
